# Association between pre-ICU statin use and ARDS mortality in the MIMIC-IV database: a cohort study

**DOI:** 10.3389/fmed.2023.1328636

**Published:** 2023-12-21

**Authors:** Hui Mao, Yi Yu, Qianqian Wang, Hengjie Li

**Affiliations:** ^1^Center for Rehabilitation Medicine, Department of Anesthesiology, Zhejiang Provincial People’s Hospital (Affiliated People’s Hospital), Hangzhou Medical College, Hangzhou, Zhejiang, China; ^2^Department of Critical Care Medicine, The Second Affiliated Hospital of Guangzhou University of Chinese Medicine, Guangzhou, Guangdong, China; ^3^Department of Pulmonary and Critical Care Medicine, The First Affiliated Hospital, Sun Yat-sen University, Guangzhou, Guangdong, China; ^4^Department of Pulmonary and Critical Care Medicine, Guangxi Hospital Division of The First Hospital, Sun Yat-sen University, Nanning, China; ^5^Emergency and Critical Care Center, Department of Emergency Medicine, Zhejiang Provincial People’s Hospital (Affiliated People’s Hospital), Hangzhou Medical College, Hangzhou, Zhejiang, China

**Keywords:** ICU, Medical Information Mart for Intensive Care-IV, statins, cohort study, mortality

## Abstract

**Background:**

Acute respiratory distress syndrome (ARDS) is a severe condition associated with high morbidity, mortality, and healthcare costs. Despite extensive research, treatment options for ARDS are suboptimal.

**Methods:**

This study encompassed patients diagnosed with ARDS from the Medical Information Mart for Intensive Care-IV (MIMIC-IV) database. Pre-intensive care unit (ICU) statin use was assessed as the exposure variable. Kaplan–Meier survival analysis was conducted to evaluate mortality at 30 and 90 days. Adjusted multivariable Cox models were utilized to estimate hazard ratios. Subgroup analyses and propensity score-matching (PSM) were undertaken for further validation.

**Results:**

Our study comprised 10,042 participants diagnosed with ARDS, with an average age of 61.8 ± 15.3 years. Kaplan–Meier survival analysis demonstrated a significantly lower prevalence of mortality at 30 and 90 days in individuals who used statins before ICU admission. Adjusted multivariable Cox models consistently showed a significant decrease in mortality prevalence associated with pre-ICU statin use. After accounting for confounding factors, patients who used statins before ICU admission experienced a 39% reduction in 30-day mortality and 38% reduction in 90-day mortality. We found a significant decrease in ICU stay (0.84 days) for those who used statins before ICU admission. These results were supported by subgroup analyses and PSM.

**Conclusion:**

This large cohort study provides evidence supporting the association between pre-ICU statin use, reduced risk of death, and shorter ICU stay in patients with ARDS, thereby suggesting the potential benefits of statin use in critically ill patients.

## Introduction

Acute respiratory distress syndrome (ARDS) is characterized by severe hypoxemic respiratory failure, with bilateral infiltrates evident on chest imaging, which is not fully explained by heart failure or fluid overload. ARDS is defined by the Berlin criteria (1). Despite significant advancements in the understanding of ARDS since its initial description over 50 years ago (2), treatments primarily offer supportive care rather than a definitive cure, which has led to a prevalence of mortality of up to 45% (3). Furthermore, survivors of ARDS often experience long-term physical, neuropsychiatric, and neurocognitive impairments that affect their quality of life significantly (4). Consequently, there is an immediate and pressing need to identify innovative and efficacious therapies to improve clinical outcomes and address the wide-ranging health consequences associated with ARDS.

Inhibitors of hydroxymethylglutaryl-coenzyme A reductase (statins) are often recommended for primary and secondary prevention of cardiovascular diseases because of their lipid-lowering properties. Recent research has highlighted the crucial anti-inflammatory, immunomodulatory, and antioxidant properties of statins (5, 6). The diverse impacts of statins have attracted growing interest across various medical disciplines, including their potential role in ARDS management. These effects occur primarily at the transcriptional level, which leads to a decrease in the synthesis of cytokines, chemokines, and C-reactive protein (7, 8). A clinical trial comparing rosuvastatin to a placebo found no significant differences in the prevalence of mortality or ventilator-free days (VFDs) (9). Another study evaluating the effects of simvastatin on VFDs and mortality did not show improved clinical outcomes. Nevertheless, the use of statin therapy in ARDS patients appears to be safe with minimal adverse effects, though the exact clinical benefits in this specific population remain uncertain (10, 11). The application of statins in ARDS treatment is controversial (12–14).

We wished to investigate the potential therapeutic benefits of pre-intensive care unit (ICU) statin use in relation to the prognosis of patients with ARDS. A retrospective analysis was conducted on a cohort of 10,042 critically ill patients using data from the MIMIC-IV dataset spanning the period from 2001 to 2019. We aimed to test the hypothesis that pre-ICU statin use in patients suffering from ARDS would be associated with a reduced prevalence of mortality and shorter stay in the ICU.

## Methods

All study data were obtained exclusively from the MIMIC-IV database (version 2.2).^1^ The MIMIC database contains information from the electronic medical records of the Beth Israel Deaconess Medical Center (Boston, MA, USA): basic demographics, laboratory results, treatment prescriptions, and records on ICU monitoring (15). The extraction process employed the Structured Query Language Server to ensure systematic retrieval of relevant data. Before accessing the database, Yi Yu secured official permission (certificate ID: 6477678) for utilization. The preparation of this report adhered strictly to the Guidelines for Strengthening the Reporting of Observational Studies in Epidemiology (16).

### Study population and data extraction

Patients included in the present study met the diagnostic criteria for ARDS according to the latest internationally recognized definition (17), the fulfillment of the criteria depends on the indicators present on the day of admission to the ICU. Only adult individuals aged ≥18 years were eligible for inclusion. If patients had multiple admissions to the ICU, only data from their initial ICU admission were considered. Patients with incomplete records of respiratory-support parameters, blood-gas analyses, and vital signs were excluded from the study. Comprehensive data collection encompassed patient demographics, vital signs, comorbidities, laboratory results, scores for clinical severity, treatments (including ventilation, administration of vasoactive drugs, and dialysis), as well as other relevant admission data.

### Use of statins

To identify the pre-ICU use of statins, prescriptions for statins before ICU admission were identified by analyzing the medication records in the MIMIC-IV database.

### Outcomes and covariates

The primary endpoints assessed were mortality at 30 and 90 days. The secondary outcomes of interest were the duration of ICU stay. The variables considered in the analysis encompassed demographic factors such as sex, age, ethnicity, marital status, and insurance status. In addition, physiological measures were included: body mass index (BMI), ARDS severity, utilization of mechanical ventilation, administration of vasoactive medications, continuous renal replacement therapy (CRRT), results of blood-gas analysis, respiratory rate, oxygen saturation in blood, mean arterial blood pressure, heart rate, white blood cell count, platelet count, as well as levels of hemoglobin, creatinine, and glucose. We collected data on disease-severity scores (Charlson, Sequential Organ Failure Assessment (SOFA), Simplified Acute Physiology Score (SAPS) II) as well as information on comorbidities.

### Statistical analyses

Appropriate statistical methods were employed to analyze data and establish a comparison of variables. Quantitative variables were summarized using interquartile ranges (IQRs) and median values, with categorical variables presented as percentages and counts. The Kruskal–Wallis test was used to determine a significant variation in continuous variables between groups given statin therapy. The chi-square test was employed to compare categorical variables. Missing data were handled by replacement with the median value. This approach was selected due to the low percentage of lack of information (0.3–4%) for height and weight variables. To ensure data completeness and increase the accuracy of results, multiple imputation methods were used to handle missing values (5–10%) in the Cox regression analysis and model building. We conducted multiple imputation by employing the R MI procedure using a chained equation approach method and 5 replications to address the missing data.

To investigate the potential impact of previous statin use on the prevalence of mortality within 30-day and 90-day timeframes, we conducted univariate and multivariate Cox regression analyses while adjusting for the aforementioned covariates. To visualize survival trends, Kaplan–Meier curves were generated and compared using the log-rank test. Furthermore, subgroup analyses were undertaken to examine the consistency of our findings across different subgroups, including age (<60 and ≥ 60 years), BMI (<25 and ≥ 25 kg/m^2^), sex (male and female), ARDS severity (mild, moderate, severe), ethnicity (white, others), SOFA score (<6 and ≥ 6), and lactate level (<4 and ≥ 4 mmol/L), with interaction effects being assessed. Propensity-score matching (PSM) was employed to enhance the robustness of our results using a 1:1 nearest neighbor-matching algorithm with a caliper width of 0.01. The hazard ratio (HR) for 30-day and 90-day mortality was estimated using a multivariable Cox proportional hazards regression model with a robust variance estimator. *P* < 0.05 (two-sided) was considered significant.

Statistical analyses were conducted using STATA 17.0 (College Station, TX, USA). This program provided the necessary tools and functions for the manipulation and modeling of data. In addition, we utilized packages from R (R Institute for Statistical Computing, Vienna, Austria) and Free Statistics 1.8 to augment analyses by leveraging the specific functionalities and statistical methods contained in these programs.

## Results

### Participants

**Figure 1:**
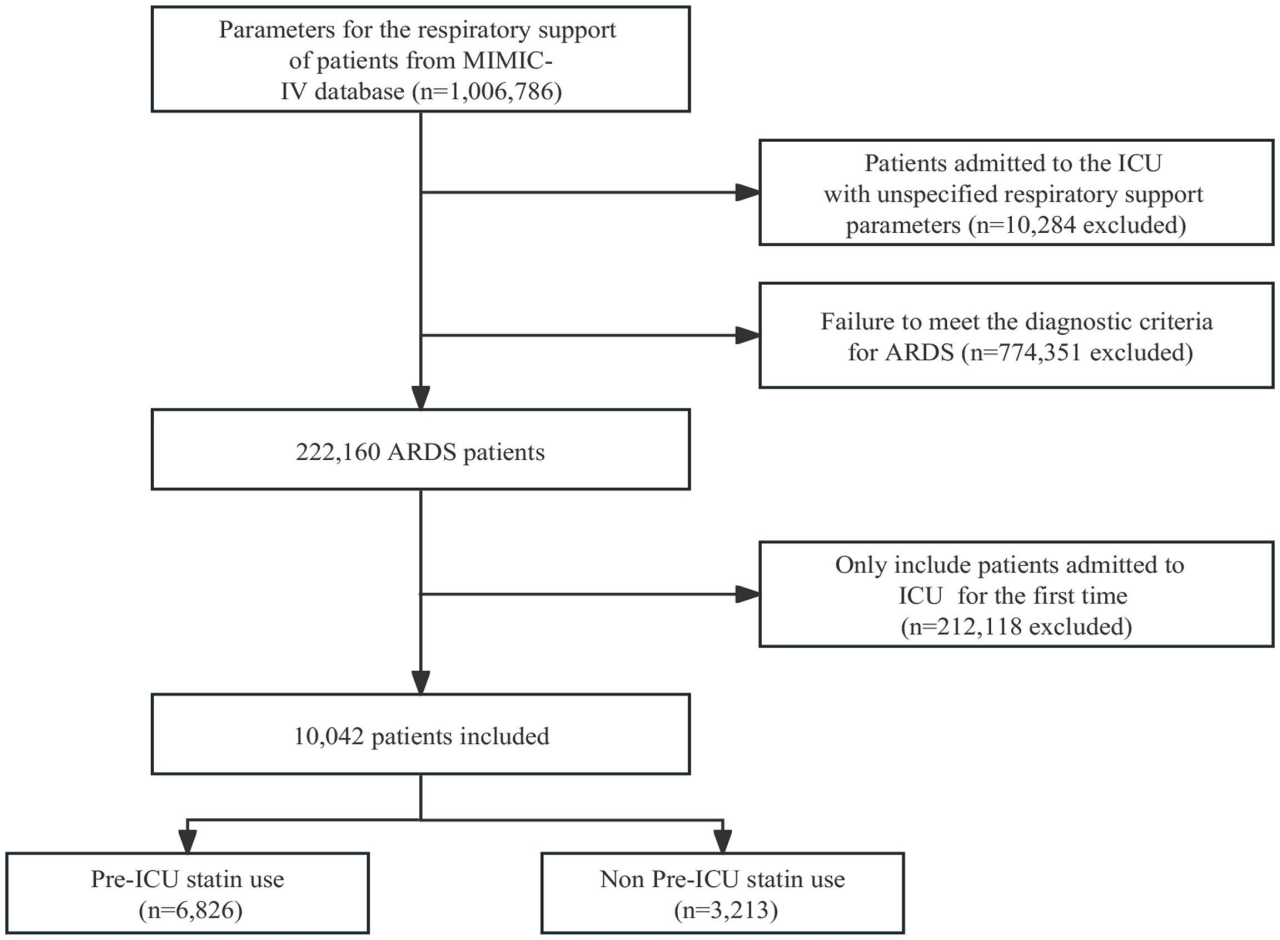
Flowchart of this study.

The flowchart illustrated in Figure 1 is a comprehensive representation of the sequential selection process employed to identify suitable study participants. Initially, all 222,160 patients underwent screening based on the predefined diagnostic criteria for ARDS. Patients with repeated admissions to the ICU were subsequently excluded from the study cohort. The final analysis was conducted on a refined cohort comprising 10,042 patients. The flowchart captures visually the systematic approach implemented to identify and select this definitive group of participants for further investigation.

### Baseline characteristics

The study cohort comprised 10,042 patients with a mean age of 61.8 ± 15.3 years, and 34.9% of the cohort were women. Table 1 provides an in-depth overview of the baseline characteristics observed within this patient population. A meticulous comparison between the two sets of data revealed conspicuous distinctions: the group who did not use statins before ICU admission tended to be younger and women. They exhibited lower levels of partial pressure of oxygen in the arterial blood/fraction of inspired oxygen (PaO_2_/FiO_2_), increased lactate level, increased use of invasive ventilator and CRRT, higher disease scores, higher prevalence of comorbid sepsis, and noticeably increased prevalence of 30-day and 90-day mortality. In contrast, the group who used statins before ICU admission statin demonstrated a lower prevalence of vasoactive-drug administration and fewer comorbidities. Supplementary Table S1 presents a comparison of baseline data between the two groups following Propensity-score matching (PSM).

**Table 1:** Characteristics of participants at baseline.

Variable	Total (*n* = 10,042)	Non pre-ICU statin use (*n* = 6,829)	Pre-ICU statin use (*n* = 3,213)	*p*
Age, years	61.8 ± 15.3	59.2 ± 16.4	67.3 ± 11.0	<0.001
Sex, female, n (%)	3,507 (34.9)	2,577 (37.7)	930 (28.9)	<0.001
BMI, kg/m^2^	29.6 ± 6.9	29.5 ± 7.2	29.7 ± 6.1	0.3
Ethnicity, n (%)				<0.001
White	6,257 (62.3)	4,047 (59.3)	2,210 (68.8)	
Other	3,785 (37.7)	2,782 (40.7)	1,003 (31.2)	
Insurance type, n (%)				<0.001
Medicaid	802 (8.0)	652 (9.5)	150 (4.7)	
Medicare	3,770 (37.5)	2,311 (33.8)	1,459 (45.4)	
Other	5,470 (54.5)	3,866 (56.6)	1,604 (49.9)	
Heart rate (bpm)	87.0 ± 15.2	88.4 ± 16.3	84.1 ± 12.2	<0.001
MAP (mmHg)	76.7 ± 9.0	77.4 ± 9.7	75.2 ± 7.3	<0.001
Respiration rate (bpm)	19.4 ± 4.0	19.8 ± 4.2	18.5 ± 3.3	<0.001
Temperature (°C)	36.9 ± 0.7	36.9 ± 0.8	36.8 ± 0.5	<0.001
SpO_2_ (%)	97.1 ± 2.8	97.0 ± 3.0	97.4 ± 2.0	<0.001
Glucose (mmol/L)	142.4 ± 45.2	144.2 ± 49.3	138.7 ± 34.9	<0.001
pH	7.4 ± 0.1	7.3 ± 0.1	7.4 ± 0.1	<0.001
PO_2_ (mmHg)	187.6 ± 75.3	173.8 ± 75.1	216.9 ± 67.1	<0.001
PCO_2_ (mmHg)	42.8 ± 9.1	43.1 ± 9.7	42.0 ± 7.5	<0.001
PaO_2_/FiO_2_	245.8 ± 95.8	241.5 ± 97.2	255.1 ± 92.0	<0.001
Lactate (mmol/L)	2.0 (1.5, 2.8)	2.0 (1.5, 3.0)	1.9 (1.5, 2.5)	<0.001
WBC count (×10^9^)	13.8 ± 9.9	13.9 ± 10.2	13.5 ± 9.4	0.045
Hb (g/L)	10.8 ± 1.9	10.9 ± 2.1	10.4 ± 1.6	<0.001
Platelets (×10^9^)	190.0 ± 100.6	193.3 ± 105.4	183.0 ± 89.3	<0.001
BUN (mg/dL)	22.3 ± 17.6	23.2 ± 18.8	20.5 ± 14.6	<0.001
Scr (mg/dL)	0.9 (0.7, 1.3)	0.9 (0.7, 1.3)	0.9 (0.8, 1.2)	0.005
Sodium (mmol/L)	138.7 ± 4.4	138.9 ± 4.8	138.2 ± 3.4	<0.001
Potassium (mmol/L)	4.3 ± 0.6	4.3 ± 0.6	4.4 ± 0.5	<0.001
Vasoactive drugs, n (%)	6,672 (66.4)	4,318 (63.2)	2,354 (73.3)	<0.001
CRRT, n (%)	634 (6.3)	533 (7.8)	101 (3.1)	<0.001
Ventilation, n (%)	8,648 (86.1)	6,115 (89.5)	2,533 (78.8)	<0.001
MI, n (%)	1,649 (16.4)	668 (9.8)	981 (30.5)	<0.001
CHF, n (%)	217 (2.2)	139 (2)	78 (2.4)	0.207
CBVD, n (%)	1,456 (14.5)	940 (13.8)	516 (16.1)	0.002
CPD, n (%)	2,360 (23.5)	1,594 (23.3)	766 (23.8)	0.582
Rheumatic disease, n (%)	275 (2.7)	184 (2.7)	91 (2.8)	0.693
Diabetes without complication, n (%)	2,223 (22.1)	1,264 (18.5)	959 (29.8)	<0.001
Diabetes with complication, n (%)	747 (7.4)	353 (5.2)	394 (12.3)	<0.001
Renal disease, n (%)	1,402 (14.0)	797 (11.7)	605 (18.8)	<0.001
Malignant cancer, n (%)	1,275 (12.7)	935 (13.7)	340 (10.6)	<0.001
Severe liver disease, n (%)	744 (7.4)	679 (9.9)	65 (2)	<0.001
Charlson comorbidity index	5.1 ± 2.8	4.8 ± 2.8	5.8 ± 2.5	<0.001
SOFA score	6.8 ± 3.4	7.1 ± 3.7	6.2 ± 2.7	<0.001
SAPS II score	41.0 ± 15.2	41.6 ± 15.8	39.6 ± 13.5	<0.001
Sepsis, n (%)	6,994 (69.6)	5,075 (74.3)	1919 (59.7)	<0.001
30-day mortality, n (%)	1,729 (17.2)	1,442 (21.1)	287 (8.9)	<0.001
90-day mortality, n (%)	1,833 (18.3)	1,527 (22.4)	306 (9.5)	<0.001
ICU stay, days	2.6 (1.3, 5.8)	3.5 (1.7, 7.9)	2.2 (1.3, 4.2)	<0.001

For each variable, mean ± standard deviation, median (interquartile range), or number (percent) was reported (as appropriate).

BMI, body mass index; MAP, mean arterial pressure; SpO_2_, pulse oxygen saturation; PH, potential of hydrogen; PO_2_, partial pressure of oxygen; PCO_2_, partial pressure of carbon dioxide; PaO_2_/FiO_2_, arterial oxygen tension/inspired oxygen fraction; WBC, white blood cell; Hb, hemoglobin; BUN, blood urea nitrogen; Scr, serum creatinine; CRRT, continuous renal replacement therapy; MI, myocardial infarct; CHF, congestive heart failure; CBVD, cerebrovascular disease; CPD, chronic pulmonary disease; SAPS, Simplified Acute Physiology Score; SOFA, Sequential Organ Failure Assessment; ICU, intensive care unit.

### Relationship between pre-ICU statin use and mortality at 30 and 90 days

**Figure 2:**
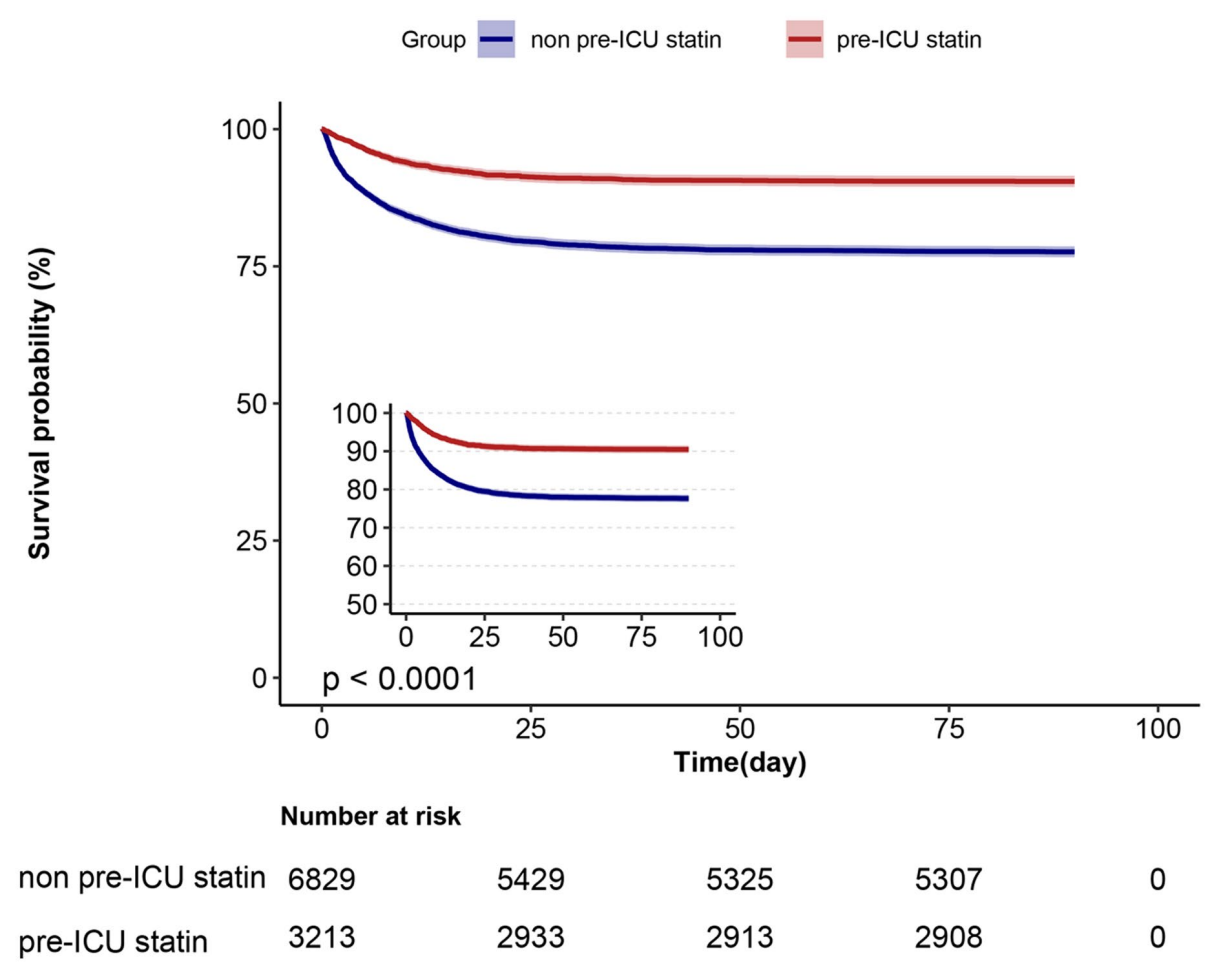
Kaplan–Meier survival curves for ICU patients at day 90 and categorized on pre-ICU statin use.

Survival analyses using the Kaplan–Meier method revealed a significant decrease in the prevalence of mortality at 30 and 90 days among patients who had pre-ICU statin use compared with those who had not (log-rank test: *p* < 0.0001) (Figure 2). In the assessment of 30-day mortality risk, pre-ICU statin use was associated with a significant reduction in death compared with non pre-ICU statin use (HR = 0.39, 95%CI = 0.34–0.44, *p* < 0.001). Consistent results were observed when analyzing data over the course of 90 days, which highlighted comparable outcomes between non pre-ICU statin use and pre-ICU statin use. A notable association between pre-ICU statin use and a lower HR was observed (HR = 0.39, 95%CI = 0.35–0.44, *p* < 0.001) (Table 2).

**Table 2:** Values of HR and 95%CI of pre-ICU statin use for 30-day mortality.

	HR	95%CI	*p*
Model 1	0.39	(0.34–0.44)	<0.001
Model 2	0.36	(0.32–0.41)	<0.001
Model 3	0.37	(0.32–0.42)	<0.001
Model 4	0.59	(0.52–0.68)	<0.001
Model 5	0.59	(0.51–0.67)	<0.001
Model 6	0.61	(0.53–0.7)	<0.001
PSM	0.74	(0.63–0.86)	<0.001

In extended multivariable Cox models (Table 2), the HRs for pre-ICU statin use remained consistently significant across all models (ranging from 0.36 to 0.61, *p* < 0.001 for all). After adjustment for the covariates listed in Table 2, we found a 39% lower risk of 30-day mortality in patients who received statins before ICU admission (HR = 0.61, 95%CI = 0.53–0.70, *p* < 0.001, model 6) (Table 2). Similarly, there was a 38% lower risk of 90-day mortality in patients who received statins before ICU admission (HR = 0.62, 95%CI = 0.54–0.71, *p* < 0.001, model 6) (Supplementary Table S2). These results indicated the robustness of the findings obtained from our models.

### Relationship between pre-ICU use of statins, duration of ICU

In the univariate analysis, the group who used statins before ICU admission had a significantly shorter ICU stay compared with those who did not use used statins before ICU admission. The reduction in ICU stay was 2.14 days (*p* < 0.001, *β* = −2.14, 95%CI = −2.43 to −1.86). This finding was consistent with the results obtained from the linear multivariate regression model. It showed a shorter ICU stay for the group who used statins before ICU admission than those who did not use statins before ICU admission. The reduction in ICU stay was 0.84 days (*p* < 0.001, *β* = −0.84, 95%CI = −1.13 to −0.55) (Table 3). The results remained significant even after using PSM to control for potential confounding variables (*p* < 0.001, *β* = −0.69, 95%CI = −1 to −0.39) (Supplementary Table S3). These results strongly suggested pre-ICU statin administration to be associated with a shorter duration of ICU stay.

**Table 3:** Pre-ICU statin use and ICU stay.

		Model 1		Model 2	
Variable	n. total	*β* (95%CI)	*p*	*β* (95%CI)	*p*
Non pre-ICU statin use	6,829	0 (Ref)		0 (Ref)	
Pre-ICU statin use	3,213	−2.14 (−2.43 to −1.86)	<0.001	−0.84 (−1.13 to −0.55)	<0.001

### Subgroup analyses and sensitivity analyses

**Figure 3:**
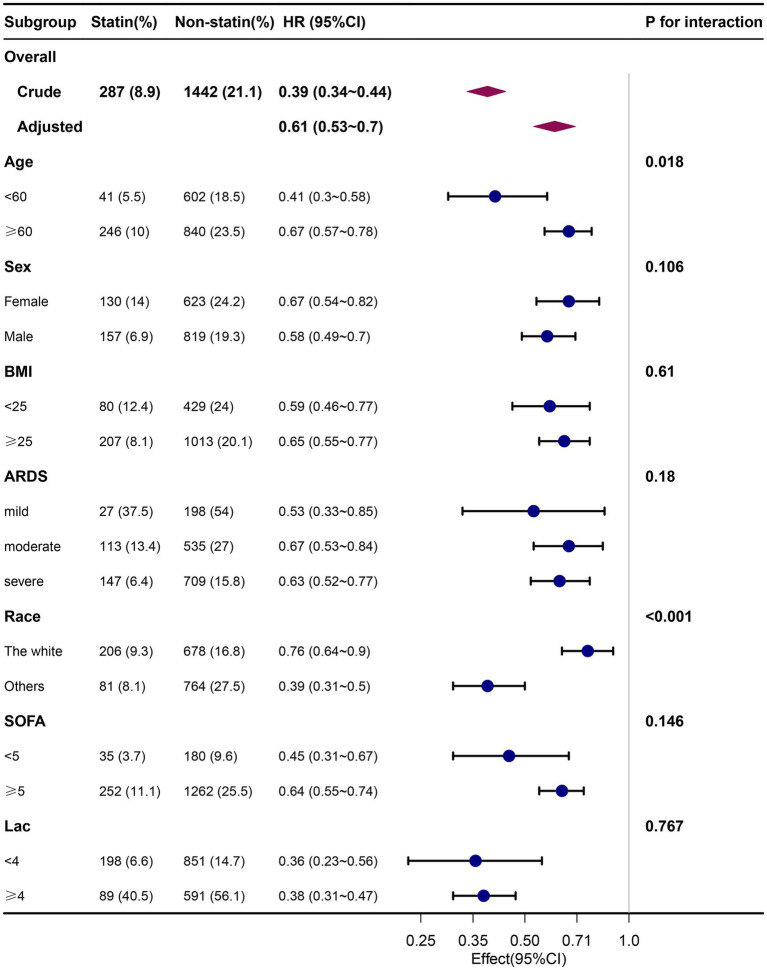
Association between pre-ICU statin use and 30-day mortality according to baseline characteristics. Each stratification adjusted for all the factors (age, sex, BMI, ethnicity, insurance, temperature, heart rate, MAP, respiration rate, SPO2, glucose, PH, PO2, PCO2, PO2/FiO2, lactate, sodium, potassium, WBC, HB, PLT, Scr, Bun, ventilation, vasoactive drugs, CRRT, SAPS II, SOFA, charlson comorbidity index, myocardial infarct, congestive heart failure, cerebrovascular disease, chronic pulmonary disease, diabetes without complication, diabetes with complication, renal disease, malignant cancer, severe liver disease, sepsis) except the stratification factor itself. BMI, body mass index; ARDS, Acute Respiratory Distress Syndrome; SOFA, Sequential Organ Failure Assessment.

Subgroup analyses showed that the relationship between pre-ICU statin use and ICU outcome remained robust and reliable. Specifically, the protective impact of pre-ICU statin use was more pronounced in patients under the age of 60 years and those of non-white ethnicity. No other significant interactions were observed in the subgroups (*p*_interaction_ > 0.05) (Figure 3).

After PSM, the study included 2,547 well-matched pairs in the group with pre-ICU statin use and the non pre-ICU statin use group. There were no significant differences between the two matched groups. Among these matched pairs in the propensity score-matched pool, patients who received statins before ICU admission had a significantly lower prevalence of mortality at 30 and 90 days (*p* < 0.001) (Supplementary Table S1).

The robustness of these findings was confirmed through Cox regression models. The univariable Cox proportional hazards regression model showed a HR of 0.74 (95%CI = 0.63–0.86, *p* < 0.0001) for 30-day mortality and a HR of 0.76 (95%CI = 0.65–0.88, *p* < 0.0001) for 90-day mortality (Table 2). These results indicated a significant reduction in the prevalence of mortality among patients who received statins before ICU admission, which suggested the beneficial effects of pre-ICU statin use on the outcome.

## Discussion

### Main findings

Our study represents the most comprehensive cohort investigation examining the impact of pre-ICU statin use on mortality in patients suffering from ARDS. We found a significant association between pre-ICU statin use and a reduction in ICU stay. Moreover, our results suggest that patients with ARDS given statins before ICU admission had a lower risk-adjusted prevalence of mortality at 30 and 90 days compared with those who did not use statins before ICU admission. Importantly, these findings remained consistent even after accounting for potential confounding factors through PSM. Our study provides strong evidence supporting the potential benefits of pre-ICU statin use in improving the outcome in patients with ARDS.

### Effects of pre-ICU statin use on mortality and ICU stay for patients suffering from ARDS

Statin administration has been associated with a reduced prevalence of mortality in individuals diagnosed with ARDS (18, 19). However, those studies used an outdated definition of ARDS. Therefore, the findings from those studies may not be entirely consistent with the current understanding of ARDS. Hence, we aimed to expand on those previous findings by specifically examining the relationship between pre-ICU statin use and death for patients diagnosed according to the latest definition of ARDS.

A retrospective study conducted in multiple medical centers explored the use of statins in patients with coronavirus disease 2019 and found a potential reduction in the risk of developing ARDS (odds ratio = 0.78, 95%CI = 0.69–0.89, *p* < 0.001) (20). A double-blind, randomized controlled trial conducted in the UK and Ireland by Agus and colleagues enrolled 540 intubated and mechanically ventilated patients with ARDS. They demonstrated that simvastatin was a cost-effective treatment for ARDS, resulting in significant gains in quality-adjusted life years and cost savings (21). Similarly, Mansur et al. conducted a prospective observational cohort study in Germany involving 404 patients with sepsis-associated ARDS. Their findings indicated that statin therapy improved 28-day survival exclusively in patients with severe ARDS compared with those not taking statins (88.5% vs. 62.5%, *p* = 0.0193) (19). We observed that pre-ICU statin use was associated with a significant reduction in the prevalence of mortality at 30 days and 90 days in patients with ARDS (HRs were 0.61 (95%CI = 0.53–0.70, *p* < 0.001) and 0.62 (95%CI = 0.54–0.71, *p* < 0.001), respectively). Moreover, pre-ICU statin use was linked to a reduction of ~0.84 days in ICU stay (*β* = −0.84, 95%CI = −1.13 to −0.55, *p* < 0.001).

There is conflicting evidence regarding the efficacy of statin use in reducing the risk of ARDS-related death and improving the prognosis, so additional studies are needed to establish a clearer understanding. A meta-analysis conducted by Nagendran et al. did not demonstrate a clinical benefit from initiating statin therapy in adult patients diagnosed with ARDS. However, that meta-analysis had limitations, including a small number of studies and populations, and only a subset of the included studies utilized established ARDS criteria for patient selection (22). In a cohort study by Oh and coworkers involving a Korean population, pre-ICU statin use did not lead to a significant reduction in 30-day mortality (*p* = 0.215) (23). However, that study had important limitations, including a lack of information on BMI, PaO_2_/FiO_2_, SAPS-II score, vasoactive-medication usage, CRRT, and the presence/absence of comorbid sepsis. These factors influence the prognosis of patients suffering from ARDS (1, 24).

The precise mechanisms through which statins reduce the risk of death in patients with ARDS are not known. The efficacy of statin therapy in mitigating cardiovascular risk is widely acknowledged, particularly because of deeper understanding of atherosclerosis as an inflammatory condition (25, 26). Experts have identified multiple mechanisms by which statins alleviate inflammation (27, 28) and endothelial dysfunction (29, 30). One study demonstrated a notable reduction in systemic and pulmonary inflammation, as well as indicators of damage to alveolar type-1 epithelial cells and the systemic vascular endothelium, in patients who received statins before treatment. Hence, the anti-inflammatory properties of statins may account for the beneficial prognosis observed with their employment in ARDS (31). Evidence suggests that simvastatin diminishes the pulmonary inflammatory response to endotoxins in healthy individuals (32). Furthermore, statins exhibit antithrombotic advantages (33, 34), as validated by a well-designed, sizable, randomized controlled trial (35, 36).

### Strengths of our study

Our study possessed four main strengths. First, we utilized a comprehensive and publicly available database, thereby ensuring the reliability and comprehensiveness of our data. The diagnostic criteria used for ARDS were current and well-defined, which enhanced the accuracy and validity of our study. Second, no study has specifically examined the impact of pre-ICU statin use on the risk of death in patients suffering from ARDS. Our findings provide clear and conclusive evidence that pre-ICU statin use reduces ICU stay significantly and lowers the prevalence of 30-day and 90-day mortality among individuals with ARDS. Third, we employed multiple regression analysis and conducted PSM to establish the robustness and reliability of our study findings. This rigorous analytical approach further strengthens the credibility and internal validity of our results. Fourth, considering the widespread use of statins for primary and secondary prevention of cardiovascular diseases, our findings hold implications and generalizability beyond the ARDS population specifically.

### Limitations of our study

Our study had five main limitations. First, ARDS is not a specific disease but instead a syndrome diagnosed based on various clinical and physiological criteria. Patients with ARDS may have different underlying risk factors, complex premorbidities and comorbidities, and potentially diverse pathophysiology (37, 38). However, this shortcoming was offset by the large number of patients enrolled in our study. Second, caution should be exercised when generalizing the findings of our study because it was conducted using data from a single ICU in the USA. The sample size was substantial and fairly representative, but conducting a multicenter prospective study in the future would be valuable to confirm the generalizability of our results. Third, the generalizability of our findings to other causes of ARDS, such as trauma, aspiration, and transfusion, is limited because >69% of patients in our cohort had sepsis-induced ARDS. Nevertheless, further analysis of this specific subset is crucial because sepsis remains the most common cause of ARDS. Fourth, this study was observational, and therefore, did not apply the optimal methodology for evaluating the effects of a drug. A future randomized controlled trial would be more appropriate. Nonetheless, our study provides a foundation for further examination of pre-ICU statin use in ARDS. Nonetheless, the limitations of our study can be mitigated partially by the considerable number of participants involved and adoption of PSM methodology. Fifth, our primary objective was to assess the influence of different types and doses of statins on the prognosis of patients with ARDS. However, the limited sample size in each group resulted in insufficient statistical power, which affected statistical outcomes significantly.

## Conclusion

Application of statins before ICU admission in patients with ARDS showed potential clinical advantages. Pre-ICU statin use significantly reduced the prevalence of mortality at 30 and 90 days in patients with ARDS, and led to a shortened ICU stay. These findings suggest the efficacy of statins in the treatment of ARDS. Statin therapy may be a feasible therapeutic choice for ARDS patients and merits further research.

## Data availability statement

Publicly available datasets were analyzed in this study. This data can be found here: the MIMIC-IV database (version 2.2; https://mimic.mit.edu/).

## Ethics statement

The studies involving humans were approved by the research, which incorporated human participants, underwent comprehensive scrutiny and obtained ethical approval from both the Massachusetts Institute of Technology and the Beth Israel Deaconess Medical Center. The studies were conducted in accordance with the local legislation and institutional requirements. The participants provided their written informed consent to participate in this study. Written informed consent was obtained from the individual(s) for the publication of any potentially identifiable images or data included in this article.

## Author contributions

HM: Writing – review & editing. YY: Writing – original draft. QW: Writing – review & editing. HL: Writing – review & editing.

## Footnotes

^1^ https://mimic.mit.edu/
