# Supplementary material for: Association between pre-ICU statin use and ARDS mortality in the MIMIC-IV database: a cohort study
Source: Front Med (Lausanne). 2023 Dec 21;10:1328636. doi: 10.3389/fmed.2023.1328636 (PMC10768014; doi:10.3389/fmed.2023.1328636)
Supplement: Supplementary file 1 [file Table_1.docx]

**Table 1:** Characteristics of participants at baseline

| **Variable** | **Total**  (n = 10,042) | **Non pre-ICU statin use**  (n = 6,829) | **Pre-ICU statin use**  (n = 3,213) | **P** |
| --- | --- | --- | --- | --- |
| Age, years | 61.8 ± 15.3 | 59.2 ± 16.4 | 67.3 ± 11.0 | < 0.001 |
| Sex, female, n (%) | 3,507 (34.9) | 2,577 (37.7) | 930 (28.9) | < 0.001 |
| BMI, kg/m^2^ | 29.6 ± 6.9 | 29.5 ± 7.2 | 29.7 ± 6.1 | 0.3 |
| Ethnicity, n (%) |  |  |  | < 0.001 |
| white | 6,257 (62.3) | 4,047 (59.3) | 2,210 (68.8) |  |
| other | 3,785 (37.7) | 2,782 (40.7) | 1,003 (31.2) |  |
| Insurance type, n (%) |  |  |  | < 0.001 |
| Medicaid | 802 (8.0) | 652 (9.5) | 150 (4.7) |  |
| Medicare | 3,770 (37.5) | 2,311 (33.8) | 1,459 (45.4) |  |
| Other | 5,470 (54.5) | 3,866 (56.6) | 1,604 (49.9) |  |
| Heart rate (bpm) | 87.0 ± 15.2 | 88.4 ± 16.3 | 84.1 ± 12.2 | < 0.001 |
| MAP (mmHg) | 76.7 ± 9.0 | 77.4 ± 9.7 | 75.2 ± 7.3 | < 0.001 |
| Respiration rate (bpm) | 19.4 ± 4.0 | 19.8 ± 4.2 | 18.5 ± 3.3 | < 0.001 |
| Temperature (℃) | 36.9 ± 0.7 | 36.9 ± 0.8 | 36.8 ± 0.5 | < 0.001 |
| SpO_2_ (%) | 97.1 ± 2.8 | 97.0 ± 3.0 | 97.4 ± 2.0 | < 0.001 |
| Glucose (mmol/L) | 142.4 ± 45.2 | 144.2 ± 49.3 | 138.7 ± 34.9 | < 0.001 |
| pH | 7.4 ± 0.1 | 7.3 ± 0.1 | 7.4 ± 0.1 | < 0.001 |
| PO_2_ (mmHg) | 187.6 ± 75.3 | 173.8 ± 75.1 | 216.9 ± 67.1 | < 0.001 |
| PCO_2_ (mmHg) | 42.8 ± 9.1 | 43.1 ± 9.7 | 42.0 ± 7.5 | < 0.001 |
| PaO_2_/FiO_2_ | 245.8 ± 95.8 | 241.5 ± 97.2 | 255.1 ± 92.0 | < 0.001 |
| Lactate (mmol/L) | 2.0 (1.5, 2.8) | 2.0 (1.5, 3.0) | 1.9 (1.5, 2.5) | < 0.001 |
| WBC count (×10^9^ ) | 13.8 ± 9.9 | 13.9 ± 10.2 | 13.5 ± 9.4 | 0.045 |
| Hb (g/L) | 10.8 ± 1.9 | 10.9 ± 2.1 | 10.4 ± 1.6 | < 0.001 |
| Platelets (×10^9^) | 190.0 ± 100.6 | 193.3 ± 105.4 | 183.0 ± 89.3 | < 0.001 |
| BUN (mg/dL) | 22.3 ± 17.6 | 23.2 ± 18.8 | 20.5 ± 14.6 | < 0.001 |
| Scr (mg/dL) | 0.9 (0.7, 1.3) | 0.9 (0.7, 1.3) | 0.9 (0.8, 1.2) | 0.005 |
| Sodium (mmol/L) | 138.7 ± 4.4 | 138.9 ± 4.8 | 138.2 ± 3.4 | < 0.001 |
| Potassium (mmol/L) | 4.3 ± 0.6 | 4.3 ± 0.6 | 4.4 ± 0.5 | < 0.001 |
| Vasoactive drugs, n (%) | 6672 (66.4) | 4318 (63.2) | 2354 (73.3) | < 0.001 |
| CRRT, n (%) | 634 (6.3) | 533 (7.8) | 101 (3.1) | < 0.001 |
| Ventilation, n (%) | 8648 (86.1) | 6115 (89.5) | 2533 (78.8) | < 0.001 |
| MI, n (%) | 1649 (16.4) | 668 (9.8) | 981 (30.5) | < 0.001 |
| CHF, n (%) | 217 (2.2) | 139 (2) | 78 (2.4) | 0.207 |
| CBVD, n (%) | 1456 (14.5) | 940 (13.8) | 516 (16.1) | 0.002 |
| CPD, n (%) | 2360 (23.5) | 1594 (23.3) | 766 (23.8) | 0.582 |
| Rheumatic disease, n (%) | 275 (2.7) | 184 (2.7) | 91 (2.8) | 0.693 |
| Diabetes without complication, n (%) | 2223 (22.1) | 1264 (18.5) | 959 (29.8) | < 0.001 |
| Diabetes with complication, n (%) | 747 (7.4) | 353 (5.2) | 394 (12.3) | < 0.001 |
| Renal disease, n (%) | 1402 (14.0) | 797 (11.7) | 605 (18.8) | < 0.001 |
| Malignant cancer, n (%) | 1275 (12.7) | 935 (13.7) | 340 (10.6) | < 0.001 |
| Severe liver disease, n (%) | 744 (7.4) | 679 (9.9) | 65 (2) | < 0.001 |
| Charlson Comorbidity Index | 5.1 ± 2.8 | 4.8 ± 2.8 | 5.8 ± 2.5 | < 0.001 |
| SOFA score | 6.8 ± 3.4 | 7.1 ± 3.7 | 6.2 ± 2.7 | < 0.001 |
| SAPS II score | 41.0 ± 15.2 | 41.6 ± 15.8 | 39.6 ± 13.5 | < 0.001 |
| Sepsis, n (%) | 6994 (69.6) | 5075 (74.3) | 1919 (59.7) | < 0.001 |
| 30-day mortality, n (%) | 1729 (17.2) | 1442 (21.1) | 287 (8.9) | < 0.001 |
| 90-day mortality, n (%) | 1833 (18.3) | 1527 (22.4) | 306 (9.5) | < 0.001 |
| ICU stay, days | 2.6 (1.3, 5.8) | 3.5 (1.7, 7.9) | 2.2 (1.3, 4.2) | < 0.001 |

For each variable, mean ± standard deviation, median (interquartile range), or number (percent) was reported (as appropriate).

BMI, body mass index; MAP, mean arterial pressure; SpO_2_, pulse oxygen saturation; PH, potential of hydrogen; PO_2_, partial pressure of oxygen; PCO_2_, partial pressure of carbon dioxide; PaO_2_/FiO_2,_ arterial oxygen tension/inspired oxygen fraction; WBC, white blood cell; Hb, hemoglobin; BUN, blood urea nitrogen; Scr, serum creatinine; CRRT, continuous renal replacement therapy; MI, myocardial infarct; CHF, congestive heart failure; CBVD, cerebrovascular disease; CPD, chronic pulmonary disease; SAPS, Simplified Acute Physiology Score; SOFA, Sequential Organ Failure Assessment; ICU, intensive care unit.

**Table 1S:** Characteristics of participants at baseline after PSM

| **Variables** | **Total**  (n = 5094) | **Non pre-ICU statin use**  (n = 2547) | **Pre-ICU statin use**  (n = 2547) | **P** |
| --- | --- | --- | --- | --- |
| Age, years | 67.0 ± 11.9 | 67.4 ± 12.5 | 66.5 ± 11.3 | 0.008 |
| Sex, female, n (%) | 1598 (31.4) | 813 (31.9) | 785 (30.8) | 0.398 |
| BMI, kg/m^2^ | 29.7 ± 6.5 | 29.7 ± 6.7 | 29.7 ± 6.2 | 0.951 |
| Ethnicity, n (%) |  |  |  | 0.352 |
| white | 3463 (68.0) | 1747 (68.6) | 1716 (67.4) |  |
| other | 1631 (32.0) | 800 (31.4) | 831 (32.6) |  |
| Insurance type, n (%) |  |  |  | 0.255 |
| Medicaid | 236 (4.6) | 112 (4.4) | 124 (4.9) |  |
| Medicare | 2280 (44.8) | 1168 (45.9) | 1112 (43.7) |  |
| other | 2578 (50.6) | 1267 (49.7) | 1311 (51.5) |  |
| Heart rate (bpm) | 84.5 ± 13.3 | 84.6 ± 13.8 | 84.5 ± 12.7 | 0.898 |
| MAP (mmHg) | 75.6 ± 7.9 | 75.6 ± 8.1 | 75.6 ± 7.7 | 0.864 |
| Respiration rate (bpm) | 18.7 ± 3.4 | 18.6 ± 3.4 | 18.7 ± 3.5 | 0.527 |
| Temperature (℃) | 36.8 ± 0.6 | 36.8 ± 0.6 | 36.8 ± 0.5 | 0.945 |
| SpO_2_ (%) | 97.3 ± 2.3 | 97.3 ± 2.4 | 97.3 ± 2.2 | 0.849 |
| Glucose (mmol/L) | 140.8 ± 39.0 | 141.5 ± 40.3 | 140.1 ± 37.7 | 0.218 |
| pH | 7.4 ± 0.1 | 7.4 ± 0.1 | 7.4 ± 0.1 | 0.609 |
| PO_2_ (mmHg) | 208.2 ± 71.5 | 207.5 ± 73.6 | 208.9 ± 69.3 | 0.488 |
| PCO_2_ (mmHg) | 42.4 ± 8.1 | 42.5 ± 8.1 | 42.3 ± 8.1 | 0.454 |
| PaO_2_/FiO_2_ | 254.3 ± 92.9 | 254.8 ± 92.2 | 253.9 ± 93.7 | 0.733 |
| Lactate (mmol/L) | 2.3 ± 1.6 | 2.3 ± 1.5 | 2.3 ± 1.7 | 0.821 |
| WBC count (×10^9^ ) | 13.6 ± 8.5 | 13.5 ± 6.3 | 13.6 ± 10.3 | 0.552 |
| Hb (g/L) | 10.5 ± 1.7 | 10.5 ± 1.7 | 10.5 ± 1.6 | 0.526 |
| Platelets (×10^9^) | 188.3 ± 95.6 | 190.8 ± 102.3 | 185.8 ± 88.4 | 0.061 |
| BUN (mg/dL) | 21.1 ± 15.2 | 21.1 ± 14.6 | 21.0 ± 15.7 | 0.811 |
| Scr (mg/dL) | 1.2 ± 1.0 | 1.2 ± 1.0 | 1.2 ± 1.0 | 0.999 |
| Sodium (mmol/L) | 138.3 ± 3.8 | 138.3 ± 4.0 | 138.3 ± 3.6 | 0.835 |
| Potassium (mmol/L) | 4.4 ± 0.5 | 4.4 ± 0.5 | 4.4 ± 0.5 | 0.869 |
| Vasoactive drugs, n (%) | 3631 (71.3) | 1812 (71.1) | 1819 (71.4) | 0.828 |
| CRRT, n (%) | 195 (3.8) | 102 (4) | 93 (3.7) | 0.511 |
| Ventilation, n (%) | 4154 (81.5) | 2083 (81.8) | 2071 (81.3) | 0.665 |
| MI, n (%) | 1046 (20.5) | 497 (19.5) | 549 (21.6) | 0.071 |
| CHF, n (%) | 123 (2.4) | 65 (2.6) | 58 (2.3) | 0.523 |
| CBVD, n (%) | 770 (15.1) | 381 (15) | 389 (15.3) | 0.754 |
| CPD, n (%) | 1234 (24.2) | 625 (24.5) | 609 (23.9) | 0.601 |
| Rheumatic disease, n (%) | 159 (3.1) | 88 (3.5) | 71 (2.8) | 0.171 |
| Diabetes without complication, n (%) | 1396 (27.4) | 701 (27.5) | 695 (27.3) | 0.851 |
| Diabetes with complication, n (%) | 509 (10.0) | 249 (9.8) | 260 (10.2) | 0.607 |
| Renal disease, n (%) | 832 (16.3) | 390 (15.3) | 442 (17.4) | 0.049 |
| Malignant cancer, n (%) | 636 (12.5) | 330 (13) | 306 (12) | 0.309 |
| Severe liver disease, n (%) | 112 (2.2) | 49 (1.9) | 63 (2.5) | 0.181 |
| Charlson Comorbidity Index | 5.7 ± 2.5 | 5.7 ± 2.5 | 5.6 ± 2.5 | 0.153 |
| SOFA score | 6.3 ± 2.9 | 6.3 ± 2.9 | 6.3 ± 2.9 | 0.942 |
| SAPS II score | 40.5 ± 14.0 | 40.7 ± 13.9 | 40.3 ± 14.2 | 0.282 |
| Sepsis, n (%) | 3276 (64.3) | 1645 (64.6) | 1631 (64) | 0.682 |
| 30-day mortality, n (%) | 665 (13.1) | 378 (14.8) | 287 (11.3) | < 0.001 |
| 90-day mortality, n (%) | 697 (13.7) | 391 (15.4) | 306 (12) | < 0.001 |
| ICU stay, days | 2.2 (1.3, 4.4) | 3.0 (1.4, 6.5) | 2.1 (1.3, 4.0) | < 0.001 |

For each variable, mean ± standard deviation, median (interquartile range), or number (percent) was reported (as appropriate).

BMI, body mass index; MAP, mean arterial pressure; SpO_2_, pulse oxygen saturation; PH, potential of hydrogen; PO_2_, partial pressure of oxygen; PCO_2_, partial pressure of carbon dioxide; PaO_2_/FiO_2,_ arterial oxygen tension/inspired oxygen fraction; WBC, white blood cell; Hb, hemoglobin; BUN, blood urea nitrogen; Scr, serum creatinine; CRRT, continuous renal replacement therapy; MI, myocardial infarct; CHF, congestive heart failure; CBVD, cerebrovascular disease; CPD, chronic pulmonary disease; SAPS, Simplified Acute Physiology Score; SOFA, Sequential Organ Failure Assessment; ICU, intensive care unit.

**Table 2** Values of HR and 95%CI of pre-ICU statin use for 30-day mortality

|  | **HR** | **95%CI** | **P** |
| --- | --- | --- | --- |
| **Model 1** | 0.39 | (0.34–0.44) | < 0.001 |
| **Model 2** | 0.36 | (0.32–0.41) | < 0.001 |
| **Model 3** | 0.37 | (0.32–0.42) | < 0.001 |
| **Model 4** | 0.59 | (0.52–0.68) | < 0.001 |
| **Model 5** | 0.59 | (0.51–0.67) | < 0.001 |
| **Model 6** | 0.61 | (0.53–0.7) | < 0.001 |
| **PSM** | 0.74 | (0.63–0.86) | < 0.001 |

**Table 2S** Values of HR and 95%CI of pre-ICU statin use for 90-day mortality

|  | **HR** | **95%CI** | **P** |
| --- | --- | --- | --- |
| **Model 1** | 0.39 | (0.35–0.44) | < 0.001 |
| **Model 2** | 0.36 | (0.32–0.41) | < 0.001 |
| **Model 3** | 0.37 | (0.33–0.42) | < 0.001 |
| **Model 4** | 0.59 | (0.52–0.67) | < 0.001 |
| **Model 5** | 0.59 | (0.52–0.68) | < 0.001 |
| **Model 6** | 0.62 | (0.54–0.71) | < 0.001 |
| **PSM** | 0.76 | (0.65–0.88) | < 0.001 |

HR, hazard ratio; CI, confidence interval; PSM, propensity score-matching.

**Model 1**: No adjusted.

**Model 2**: age, sex, BMI.

**Model 3: Model 2**, ethnicity, insurance.

**Model 4: Model 3**, temperature, heart rate, MAP, respiration rate, SPO_2_, glucose, pH, PO_2_, PCO_2_, PO_2_/FiO_2_, lactate, sodium, potassium, WBC count, HB, PLT, Scr, Bun.

**Model 5: Model 4**, ventilation, vasoactive drugs, CRRT, SAPS II, SOFA.

**Model 6: Model 5**, Charlson Comorbidity Index, myocardial infarct, congestive heart failure, cerebrovascular disease, chronic pulmonary disease, diabetes without complication, diabetes with complication, renal disease, malignant cancer, severe liver disease, sepsis.

**Table 3** Pre-ICU statin use and ICU stay

|  |  | **Model 1** |  | **Model 2** |  |
| --- | --- | --- | --- | --- | --- |
| Variable | n. total | β (95%CI) | P | β (95%CI) | P |
| Non pre-ICU statin use | 6,829 | 0 (Ref) |  | 0 (Ref) |  |
| pre-ICU statin use | 3,213 | −2.14 (−2.43 to −1.86) | < 0.001 | −0.84 (−1.13 to −0.55) | < 0.001 |

**Table 3S** Pre-ICU statin use for stay in the ICU after PSM

|  |  | **Model 1** |  | **Model 2** |  |
| --- | --- | --- | --- | --- | --- |
| Variable | n. total | β (95%CI) | P | β (95%CI) | P |
| Non pre-ICU statin use | 2,547 | 0 (Ref) |  | 0 (Ref) |  |
| pre-ICU statin use | 2,547 | −0.8 (−1.14 to −0.45) | <0.001 | −0.69 (−1 to −0.39) | < 0.001 |

Ref, reference; CI, confidence interval; PSM, propensity score-matching.

**Model 1** no adjustment.

**Model 2** adjusted for age, sex, BMI, ethnicity, insurance, temperature, heart rate, MAP, respiration rate, SPO_2_, glucose, PH, PO_2_, PCO_2_, PO_2_/FiO_2_, lactate, sodium, potassium, WBC, HB, PLT, Scr, Bun, ventilation, vasoactive drugs, CRRT, SAPS II, SOFA, Charlson Comorbidity Index, myocardial infarct, congestive heart failure, cerebrovascular disease, chronic pulmonary disease, diabetes without complication, diabetes with complication, renal disease, malignant cancer, severe liver disease, sepsis.
