# Supplementary material for: Association between pre-ICU statin use and ARDS mortality in the MIMIC-IV database: a cohort study
Source: Front Med (Lausanne). 2023 Dec 21;10:1328636. doi: 10.3389/fmed.2023.1328636 (PMC10768014; doi:10.3389/fmed.2023.1328636)
Supplement: Supplementary file 2 [file Table_2.docx]

**Table 2** Values of HR and 95%CI of pre-ICU statin use for 30-day mortality

|  | **HR** | **95%CI** | **P** |
| --- | --- | --- | --- |
| **Model 1** | 0.39 | (0.34–0.44) | < 0.001 |
| **Model 2** | 0.36 | (0.32–0.41) | < 0.001 |
| **Model 3** | 0.37 | (0.32–0.42) | < 0.001 |
| **Model 4** | 0.59 | (0.52–0.68) | < 0.001 |
| **Model 5** | 0.59 | (0.51–0.67) | < 0.001 |
| **Model 6** | 0.61 | (0.53–0.7) | < 0.001 |
| **PSM** | 0.74 | (0.63–0.86) | < 0.001 |

**Table 2S** Values of HR and 95%CI of pre-ICU statin use for 90-day mortality

|  | **HR** | **95%CI** | **P** |
| --- | --- | --- | --- |
| **Model 1** | 0.39 | (0.35–0.44) | < 0.001 |
| **Model 2** | 0.36 | (0.32–0.41) | < 0.001 |
| **Model 3** | 0.37 | (0.33–0.42) | < 0.001 |
| **Model 4** | 0.59 | (0.52–0.67) | < 0.001 |
| **Model 5** | 0.59 | (0.52–0.68) | < 0.001 |
| **Model 6** | 0.62 | (0.54–0.71) | < 0.001 |
| **PSM** | 0.76 | (0.65–0.88) | < 0.001 |

HR, hazard ratio; CI, confidence interval; PSM, propensity score-matching.

**Model 1**: No adjusted.

**Model 2**: age, sex, BMI.

**Model 3: Model 2**, ethnicity, insurance.

**Model 4: Model 3**, temperature, heart rate, MAP, respiration rate, SPO_2_, glucose, pH, PO_2_, PCO_2_, PO_2_/FiO_2_, lactate, sodium, potassium, WBC count, HB, PLT, Scr, Bun.

**Model 5: Model 4**, ventilation, vasoactive drugs, CRRT, SAPS II, SOFA.

**Model 6: Model 5**, Charlson Comorbidity Index, myocardial infarct, congestive heart failure, cerebrovascular disease, chronic pulmonary disease, diabetes without complication, diabetes with complication, renal disease, malignant cancer, severe liver disease, sepsis.
