# Supplementary material for: Association between pre-ICU statin use and ARDS mortality in the MIMIC-IV database: a cohort study
Source: Front Med (Lausanne). 2023 Dec 21;10:1328636. doi: 10.3389/fmed.2023.1328636 (PMC10768014; doi:10.3389/fmed.2023.1328636)
Supplement: Supplementary file 3 [file Table_3.docx]

**Table 3** Pre-ICU statin use and ICU stay

|  |  | **Model 1** |  | **Model 2** |  |
| --- | --- | --- | --- | --- | --- |
| Variable | n. total | β (95%CI) | P | β (95%CI) | P |
| Non pre-ICU statin use | 6,829 | 0 (Ref) |  | 0 (Ref) |  |
| pre-ICU statin use | 3,213 | −2.14 (−2.43 to −1.86) | < 0.001 | −0.84 (−1.13 to −0.55) | < 0.001 |

**Table 3S** Pre-ICU statin use for stay in the ICU after PSM

|  |  | **Model 1** |  | **Model 2** |  |
| --- | --- | --- | --- | --- | --- |
| Variable | n. total | β (95%CI) | P | β (95%CI) | P |
| Non pre-ICU statin use | 2,547 | 0 (Ref) |  | 0 (Ref) |  |
| pre-ICU statin use | 2,547 | −0.8 (−1.14 to −0.45) | <0.001 | −0.69 (−1 to −0.39) | < 0.001 |

Ref, reference; CI, confidence interval; PSM, propensity score-matching.

**Model 1** no adjustment.

**Model 2** adjusted for age, sex, BMI, ethnicity, insurance, temperature, heart rate, MAP, respiration rate, SPO_2_, glucose, PH, PO_2_, PCO_2_, PO_2_/FiO_2_, lactate, sodium, potassium, WBC, HB, PLT, Scr, Bun, ventilation, vasoactive drugs, CRRT, SAPS II, SOFA, Charlson Comorbidity Index, myocardial infarct, congestive heart failure, cerebrovascular disease, chronic pulmonary disease, diabetes without complication, diabetes with complication, renal disease, malignant cancer, severe liver disease, sepsis.
